# RBM15 suppresses hepatic insulin sensitivity of offspring of gestational diabetes mellitus mice via m6A-mediated regulation of CLDN4

**DOI:** 10.1186/s10020-023-00615-8

**Published:** 2023-02-20

**Authors:** Jie Fang, Xiafei Wu, Jie He, Hanwen Zhang, Xuyang Chen, Hua Zhang, Boris Novakovic, Hongbo Qi, Xinyang Yu

**Affiliations:** 1 The Department of Obstetrics, The First Affiliated Hospital of Chongqing Medical University, 1 Youyi Rd., Chongqing, 400016 China; 2 Chongqing Key Laboratory of Maternal and Fetal Medicine, Chongqing Medical University, Chongqing, 400016 China; 3 Department of Paediatrics, University of Melbourne, Royal Children’s Hospital, Parkville, VIC Australia; 4 Chongqing Health Center for Women and Children, Chongqing, 401120 China

**Keywords:** Gestational diabetes mellitus (GDM), N6-methyladenosine (m6A), Metabolic syndrome, RBM15, CLDN4, RNA modifications

## Abstract

**Background:**

Gestational diabetes Mellitus (GDM) is a common pregnancy-specific disease with high morbidity, which is linked to a high risk of obesity and diabetes in offspring. N6-methyladenosine modification of RNA is emerging as an important epigenetic mechanism that is widely manifested in many diseases. This study aimed to investigate the mechanism of m6A methylation in metabolic syndrome in offspring result from intrauterine hyperglycemia.

**Methods:**

GDM mice were established by feeding a high-fat diet 1 weeks before pregnancy. The m6A RNA methylation quantification kit was used to detect liver tissue methylation levels. PCR array was used to determine the expression of the m6A methylation modification enzyme. Immunohistochemistry, qRT-PCR, and western blot were used to examine the expression of RBM15, METTL13, IGF2BP1, and IGF2BP2. Subsequently, methylated RNA immunoprecipitation sequencing combined with mRNA sequencing, followed by dot blot and glucose uptake tests, were performed.

**Results:**

In this study, we found that offspring from a GDM mother were more vulnerable to glucose intolerance and insulin resistance. GC–MS revealed significant metabolic changes including saturated fatty acids and unsaturated fatty acids in liver of GDM offspring. We also demonstrated that global mRNA m6A methylation level was significantly increased in the fetal liver of GDM mice, indicating epigenetic change may have a strong relationship with the mechanism of metabolism syndrome. Concordantly, RBM15, the RNA binding methyltransferase, was upregulated in the liver. In vitro, RBM15 suppressed insulin sensitivity and increased insulin resistance through m6A-regulated epigenetic inhabitation of CLDN4. Moreover, MeRIP-sequencing and mRNA-sequencing revealed that differently regulated genes with differential m6A peaks were enriched in metabolic pathways.

**Conclusion:**

Our study revealed the essential role of RBM15 in insulin resistance and the effect of RBM15-regulated m6A modification in the metabolic syndrome of offspring of GDM mice.

**Supplementary Information:**

The online version contains supplementary material available at 10.1186/s10020-023-00615-8.

## Background

Gestational diabetes mellitus (GDM) is a condition in which carbohydrate intolerance develops during pregnancy (2018). With the increase of obese pregnant women, the incidence of GDM is increasing worldwide (Ferrara 2007). Fetal exposure to maternal diabetes may have a detrimental impact on the development and the function of metabolic organs through epigenetic mechanisms (Howe et al. 2020). In addition to the short-term adverse outcomes such as macrosomia and dystocia, GDM also increases the risks of adult-onset metabolic syndrome and cardiovascular diseases (2018). Insulin resistance is one of the physiological and pathological bases of metabolic syndrome and type II diabetes. A typical mechanism underlying insulin resistance is dysregulation of insulin signaling, such as improper phosphorylation of insulin receptor substrates (Copps et al. 2016; Copps and White 2012). However, the mechanism of how intrauterine hyperglycemia leads to insulin resistance and long-term complications in offspring has not been well elucidated.

Several studies have explored different molecular mechanism of epigenetic regulation involving DNA and histone modification, while RNA modification in GDM remains poorly investigated (Elliott et al. 2019). Recent studies have indicated that aberrant RNA modification is involved in different human diseases. Among all RNA modifications, N6-methyladenosine (m6A) is the most common internal modification of eukaryotic mRNA (Pinello et al. 2018). The m6A modification is reversible and dynamic in mammals, and regulated by methyltransferases (writers), demethylases (erasers), and effective proteins (readers) (Pinello et al. 2018; Jiang et al. 2021). The core methyltransferase complex consists of WTAP, METTL3 and METTL14 (Wang et al. 2016; Ping et al. 2014). Ancillary to this core structure are some m6A regulatory proteins necessary to the full methylation program, including RBM15, CBLL1, ZC3H13, and VIRMA (Patil et al. 2016, Knuckles et al. 2018, Yue et al*.* 2018, Wen et al. 2018). FTO and ALKBH5 act as demethylation enzymes to reverse methylation (Zheng et al. 2013; Jia et al. 2011). The dynamic regulation of m6A is primarily determined by the m6A methyltransferases and demethylases. Finally, to perform basic molecular functions including microRNA (miRNA) processing, mRNA transport and localization, and RNA–protein interaction, the effective proteins such as IGF2BP1-3, YTHDF1-3, YTHDC1-2, and eIF3 acted as m6A "readers" to recognize and bind to m6A (Liu et al. 2015; Alarcon et al. 2015).

Most m6A modified proteins have been shown to play vital roles in different diseases. For instance, in hepatocellular carcinoma, METTL3 is identified as a carcinoma promoter via m6A modification of SOCS2 (Chen et al. 2018), while METTL14 is a tumor suppressor by multiplying the m6A-mediated processing of pre-miR126 (Ma et al. 2017). FTO enhances invasiveness and proliferation of Gastric squamous cell carcinoma cells (Xu et al. 2017). Overexpressed ALKBH5 contributes to the maintenance and proliferation of glioblastoma stem-like cells via m6A-mediated regulation of FOXM1 (Zhang et al. 2017). In addition, METTL3-induced aberrant m6A modification possibly contributes to trophoblast dysfunction in preeclampsia (Gu et al. 2021; Su et al. 2016).

GDM-associated intrauterine hyperglycemia in the late trimester poses a danger to the fetus. Apart from affecting fetal growth and development, it possibly leads to persistent and irreversible changes to the genetic make-up of the fetus, resulting in increased susceptibility to certain long-term diseases (Su et al. 2016). In recent years, DNA methylation, histone modification, and non-coding RNA changes caused by GDM in the first filial (F1) generation have been widely investigated (Howe et al. 2020; Elliott et al. 2019). Nevertheless, few studies focus on RNA methylation. Therefore, this study was designed to investigate the effect of m6A methylation of RNA on the occurrence of long-term metabolic syndrome in offspring.

## Results

### The decreased glucose tolerance and increased insulin resistance in F1 generation of GDM

**Fig. 1:**
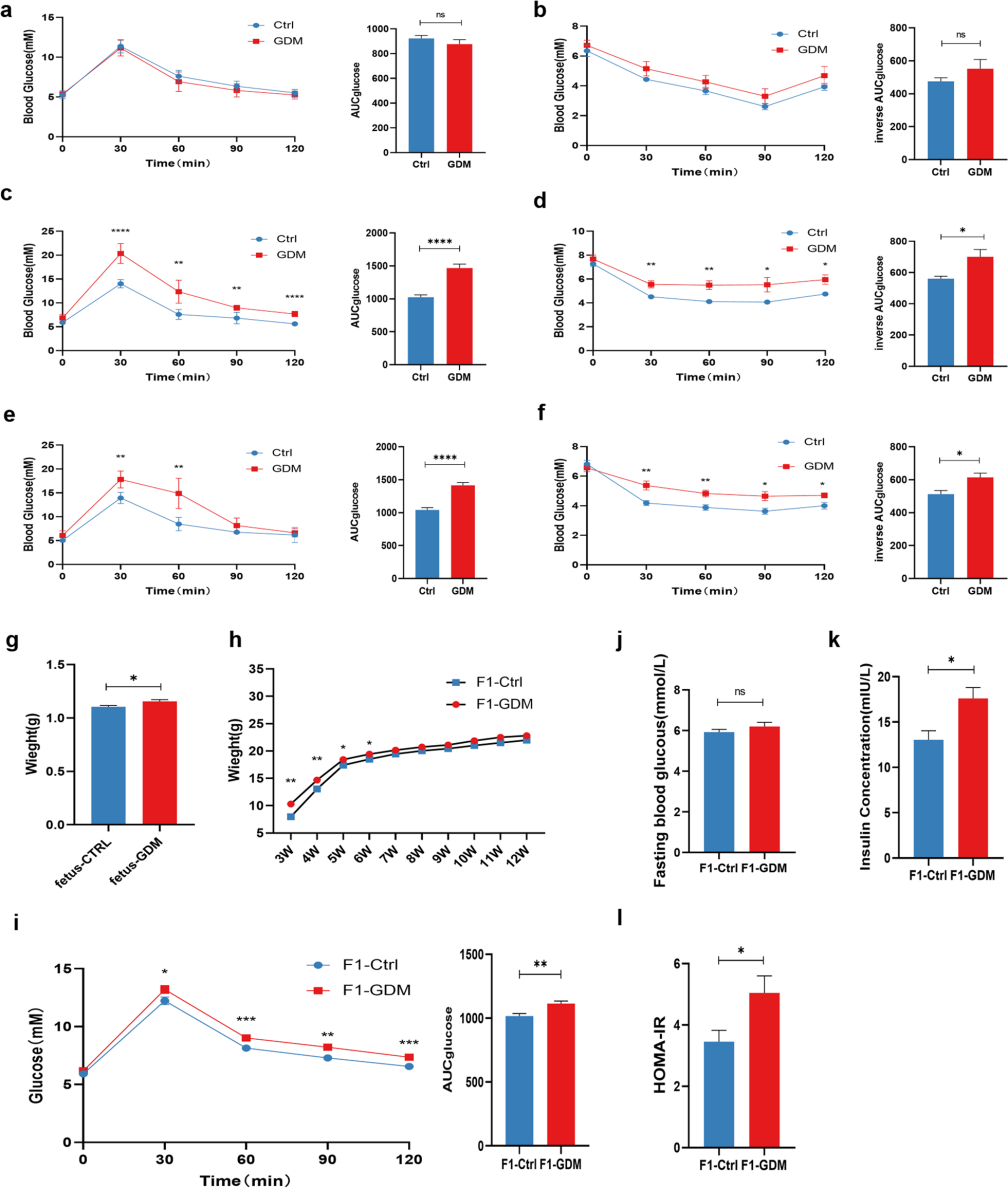
The difference of HOMA index and OGTT in F1 generation of GDM. OGTT curves for the HFD-diet group (n = 6) and control group (n = 6) of mice and comparison of areas under the curve (AUC) at gestational day (GD)0.5 (**a**), GD11.5 (**c**), and GD16.5 (**e**). ITT curves for the two groups of mice and comparison of AUC at GD0.5 (**b**), GD11.5 (**d**), and GD16.5 (**f**). **g** Fetal weight at GD18.5 of two groups. Student’s *t*-test was used and *P < 0.05. **h** Weight of F1 generation of GDM group and control from 3 to 12 weeks,*P < 0.05, **P < 0.01. **i** OGTT curves for the female offspring of two groups at the age of 12 weeks. **j** Fasting blood glucose of offspring at 12 weeks. T-test was used, P-value > 0.05. **k** Fasting insulin concentration of offspring at 12 weeks. Student’s *t*-test was used and *P < 0.05. **l** HOMA-index of offspring at 12 weeks. Student’s *t*-test was used and *P < 0.05. Results are shown as mean ± SEM

To investigate the difference of glucose tolerance and insulin resistance in the offspring of GDM, the C57BL/6J mice were fed with a high-fat diet to establish a model of gestational diabetes mellitus. AUC of OGTT and inverse AUC of ITT showed no difference between the GDM group and the controls at GD0.5 (Fig. 1a, b), while the HFD mice exhibited a less efficient tolerance of blood glucose than the control, as evidenced by the increased AUC of OGTT and inverse AUC of ITT respectively (Fig. 1c–f), which was consistent with the clinical definition of GDM. Therefore, we successfully established a GDM mice model induced by a high-fat diet.

Fetal weight was significantly increased in the GDM group at 18.5 days of gestation (Fig. 1g). The weekly weight measurement revealing that the offspring mice of GDM had a higher weight than the controls at the early stage (before 6W) (Fig. 1h). Furthermore, The OGTT performed on F1 generation demonstrated that the glucose tolerance of the GDM fetus was significantly lower, indicating less competent blood glucose clearance (Fig. 1i). Although the fasting blood glucose showed no significant difference (Fig. 1j), the serum insulin and HOMA-IR index were remarkably increased (Fig. 1k, l). Collectively, our data demonstrated that the offspring of GDM are more likely to develop insulin resistance than normal mice.

### Aberrant metabolism in F1 generation of GDM mice

**Fig. 2:**
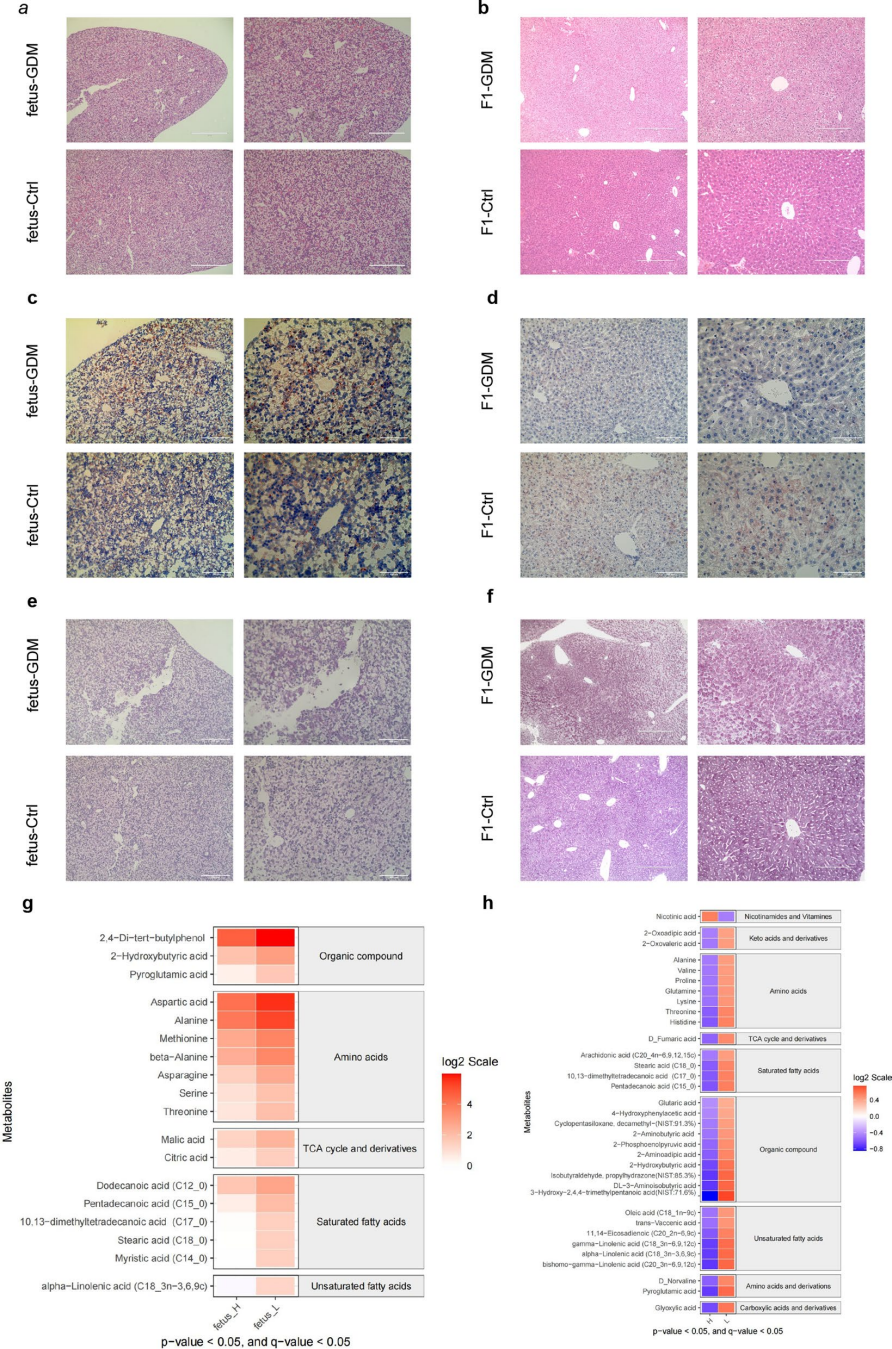
Aberrant metabolism in F1 generation of GDM mice. **a**, **b** Showed that the HE staining of GD18.5 fetus and 12-week offspring of GDM group compared with control group respectively. **c**, **d** Images of O oil red staining of GD18.5 fetus and 12-week offspring of GDM group compared with control group respectively. **e**, **f** PAS staining of two groups to confirm the presence of glycogen deposits. **g** Differentially abundant metabolites in the GD18.5 fetus of GDM compared with control determined by GC–MS (n = 6). **h** Differentially abundant metabolites of 12-week offspring of GDM compared with control (n = 6). Expression of log2 fold change relative to control mice. TCA cycle, tricarboxylic acid cycle. Scale bars is 200 μm

To identify whether there were histomorphological and metabolism-related differences, we completed a series of staining. HE staining of GD18.5 fetal liver tissue showed no visible morphological difference (Fig. 2a). Nevertheless, the images at 12-weeks fetus suggested that compared with the normal group, the structure of GDM group on liver lobules was blurred, and obvious vacuoles appeared (Fig. 2b). Notably, in the Oil Red O staining, there was no visible change in lipid droplet storage in the liver sections of mice aged 18.5 days and 12 weeks (Fig. 2c, d). Meanwhile, the cholesterol and triglyceride concentration in liver tissue and in serum showed no differences (Additional file 1: Fig. S1a–d). Periodic Acid-Schiff staining indicated no significant glycogen deposition in the two groups of GD18.5 fetal mice. In contrast, additional changes in carbohydrate metabolism were indicated by a marked increase in glycogen deposits in the 12-week F1 mice liver tissue (Fig. 2e, f).

The metabolite levels in the livers were analyzed by gas chromatography-mass spectrometry (GC–MS) to figure out whether these changes were detectable. Specifically, parts of amino acids (Serine, aspartic acid, alanine) and saturated fatty acids were significantly different at fetal stage (Fig. 2g). At the same time, the differences were mainly concentrated in the metabolism of amino acids, saturated fatty acids, and partial unsaturated fatty acids at adult stage (Fig. 2h). Stearic acid and 10,13-dimethyltetradecanoic acid, and some saturated fatty acids were the common different metabolitic between fetus and 12W offspring, which may be a long-term metabolism problem due to maternal hyperglycemia during pregnancy.

### RBM15 expression was increased in fetal liver tissue

**Fig. 3:**
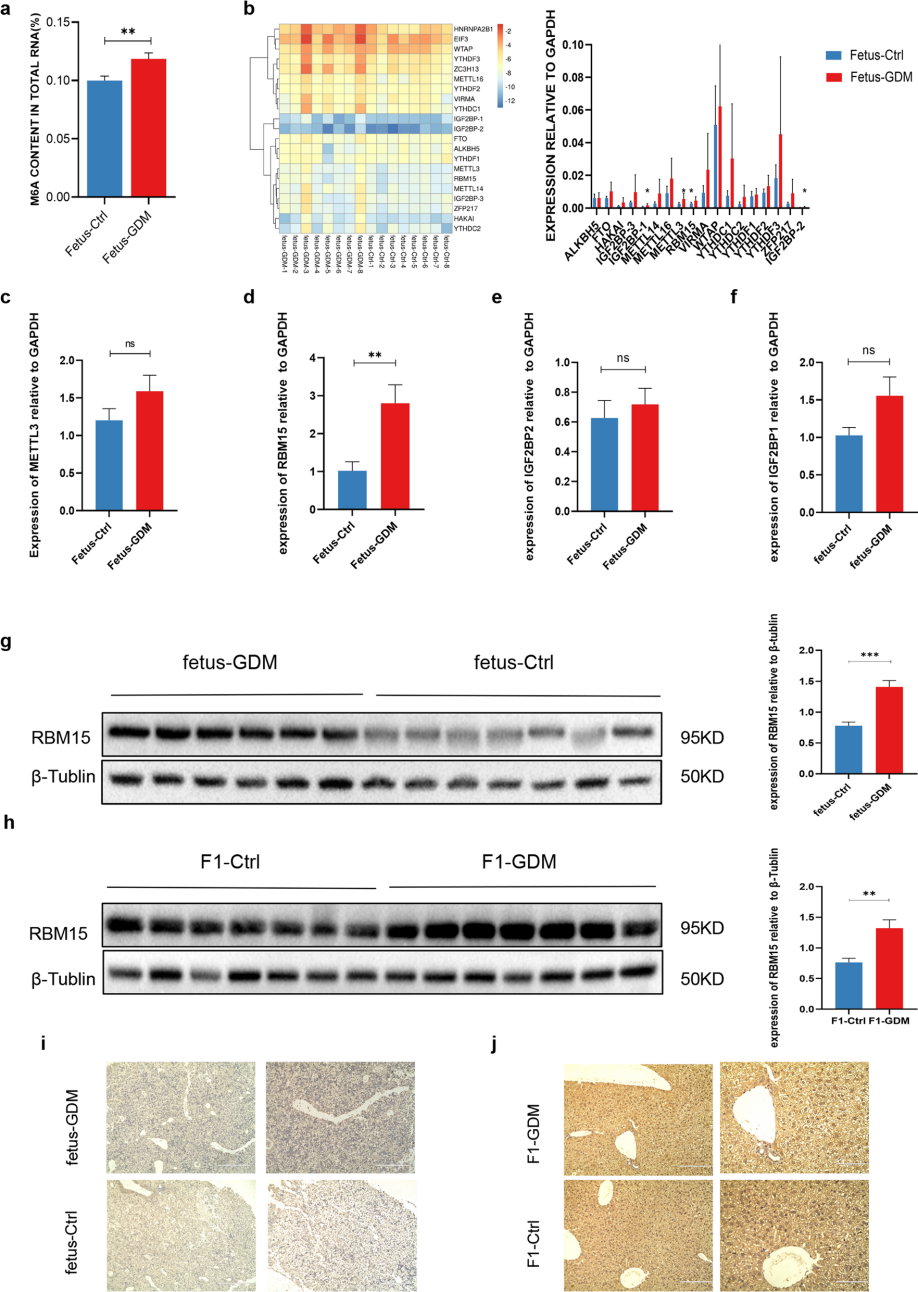
The expression of RBM15 was increased in F1 generation liver tissue. **a** The methylated RNA (m6A) level was determined in liver tissue of GD18.5 fetus of the GDM group and Control (n = 8) (**P < 0.01 by Student’s t-test); **b** PCR array of m6A methyltransferases, demethylases and effector proteins was measured in liver tissue of GDM group and Control (n = 8). Expression of log10 fold change relative to control mice. mRNA expression levels of METTL3 (**c**), RBM15 (**d**), IGF2BP1 (**e**) and IGF2BP2 (**f**) in GD18.5 fetus liver were examined using RT-qPCR (n = 12). Protein levels of both GD18.5 fetus (**g**) and F1 generation at the age of 12 weeks (**h**) were determined by western blot. **i**, **j** Image of IHC staining of RBM15 in GD18.5 fetus and 12-week offspring liver tissue respectively. Results are shown as mean ± SEM, *P < 0.05, ** P < 0.01, ***P < 0.001

To explore the mechanism of m6A modification in insulin resistance in the F1 generation, we firstly analyzed the global methylation levels in liver tissues of GD18.5 fetus. Our data showed that m6A RNA modification was significantly increased in the GDM group (Fig. 3a). In order to identify potential contributors to this increase in m6A, we used PCR Arrays to determine the expression levels of m6A-related modifier enzymes, identifying noteworthy differences of METTL3, RBM15, IGF2BP1 and IGF2BP2 (Fig. 3b). Combined with verification in 12 pairs of samples, the RBM15 that was differentially overexpressed in m6A methyltransferase "writers" was screened out as the most significant (Fig. 3c–f).

Similarly, RBM15 protein expression levels of both GD18.5 and 12-week mice liver in the GDM group were significantly higher (Fig. 3g, h, Additional file 1: Fig. S2a, b). Furthermore, immunohistochemistry was performed to determine the localization and expression of RBM15 in liver tissue. As shown in Fig. 3i and j, RBM15 was mainly located in the nucleus of the liver. Taken together, this evidence implied that the level of m6A methylation and m6A methyltransferases RBM15 were concordantly higher in fetal liver tissue of F1 mice from the GDM group compared to controls.

### General features of m6A methylation in livers of offspring of GDM mouse and controls

**Fig. 4:**
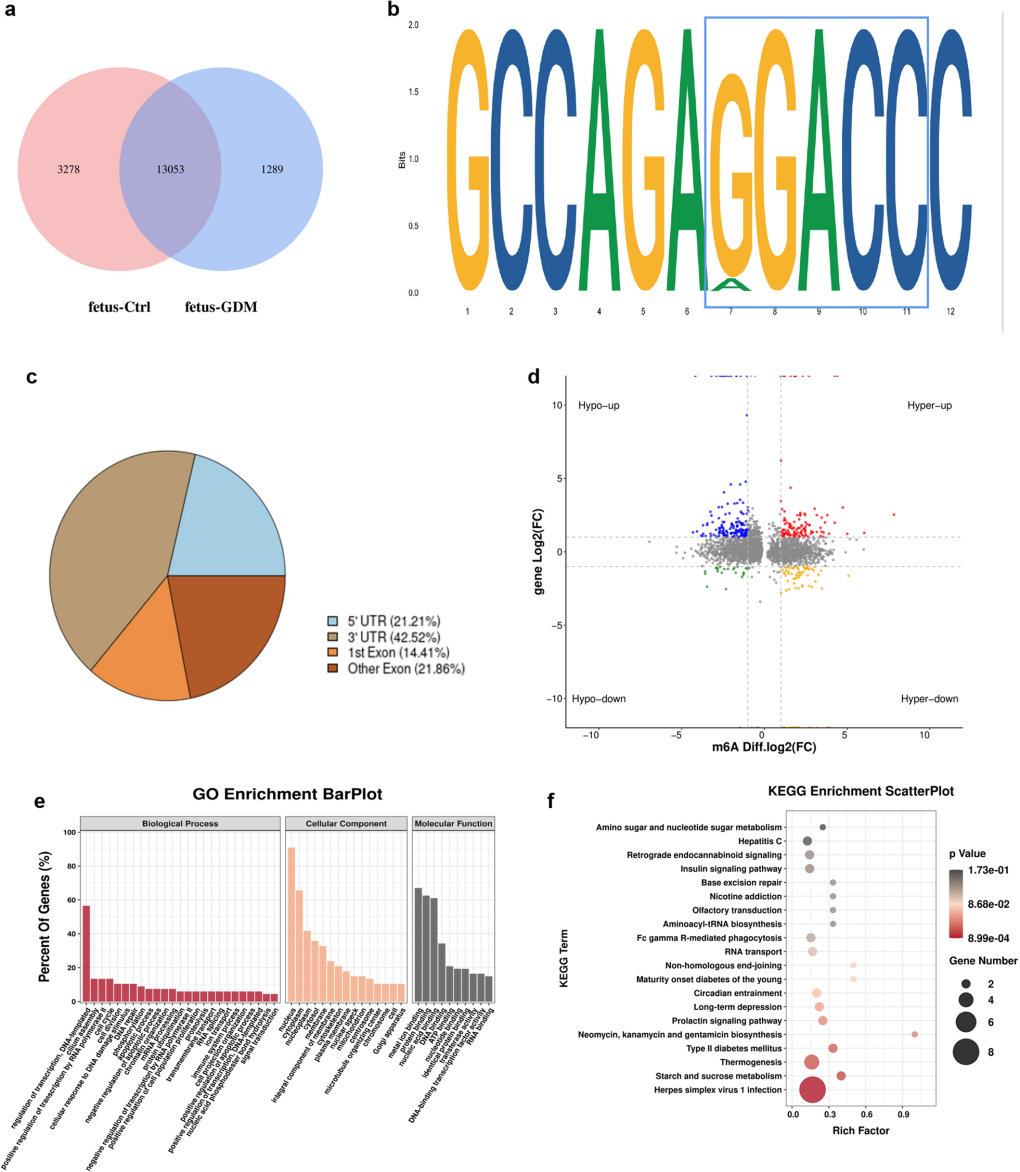
General features of m6A methylation in livers of offspring of GDM mouse and controls. **a** Venn diagram showing the overlap of m6A peaks in two groups. **b** The motif research for m6A-containing peak regions. **c** The distribution of differential Peaks on gene functional elements. **d** The starplot showed the genes with both differential (hyper or hypo) m6A peaks (Y axis; P < 0.05) and differential (up or down) expression (X axis; P < 0.05) in GDM group compared with control group. **e** The gene ontology for the hypo-up, hyper-up, hypo-down and hyper-down regulated genes. **f** Bar plot showing the top twenty enrichment scores of the significant enrichment pathway for the hypo-up, hyper-up, hypo-down and hyper-down regulated genes

Next, high-throughput sequencing (MeRIP-seq) was performed to detect genome-wide m6A RNA modifications in GD18.5 fetal livers of GDM and control mice. The MeRIP-seq analysis of feta mice livers revealed that, 13053 m6A recurrent peaks were detected in both control and GDM groups (Fig. 4a). Motif search showed that m6A peaks contained the RRACH conserved sequence motif (R represents purine, A is m6A and H is a non-guanine base), which appeared to be necessary for m6A modification, indicating the reliability of the data (Fig. 4b). Further analysis of distribution profiles of m6A peaks revealed that the most differentially methylated m6A sites (DMMSs) were within 3′UTR (Fig. 4c).

We also found 25 upregulated mRNAs with increased m6A abundance, 164 upregulated mRNAs with decreased m6A abundance, 6 downregulated mRNAs with decreased m6A abundance, and 6 downregulated mRNAs with increased m6A abundance (Fig. 4d). Differentially expressed mRNAs with increased or decreased m6A methylation were selected to perform GO enrichment analysis and KEGG pathway analysis to investigate the function m6A modification of fetus of GDM mice. For the BP category, we observed that the selected genes were mainly enriched in the regulation of transcription, such as regulation of transcription, DNA-templated, and positive transcription regulation by RNA polymerase II. For the CC category, genes with DMMs were mainly enriched in membranes and cytoplasm. While for the MF category, genes containing DMMs were mostly enriched in protein binding and metal ion binding regions (Fig. 4e). In contrast, KEGG pathway analysis revealed that selected genes were mainly enriched in some metabolism-related pathways, including type II Diabetes Mellitus, insulin signaling pathway, starch and sucrose metabolism, and maturity-onset diabetes of the young (Fig. 4f), indicating the potential possibility that those differentiated genes were associated with metabolism syndrome of the offspring of GDM.

### RBM15 enhances insulin resistance in vitro

**Fig. 5:**
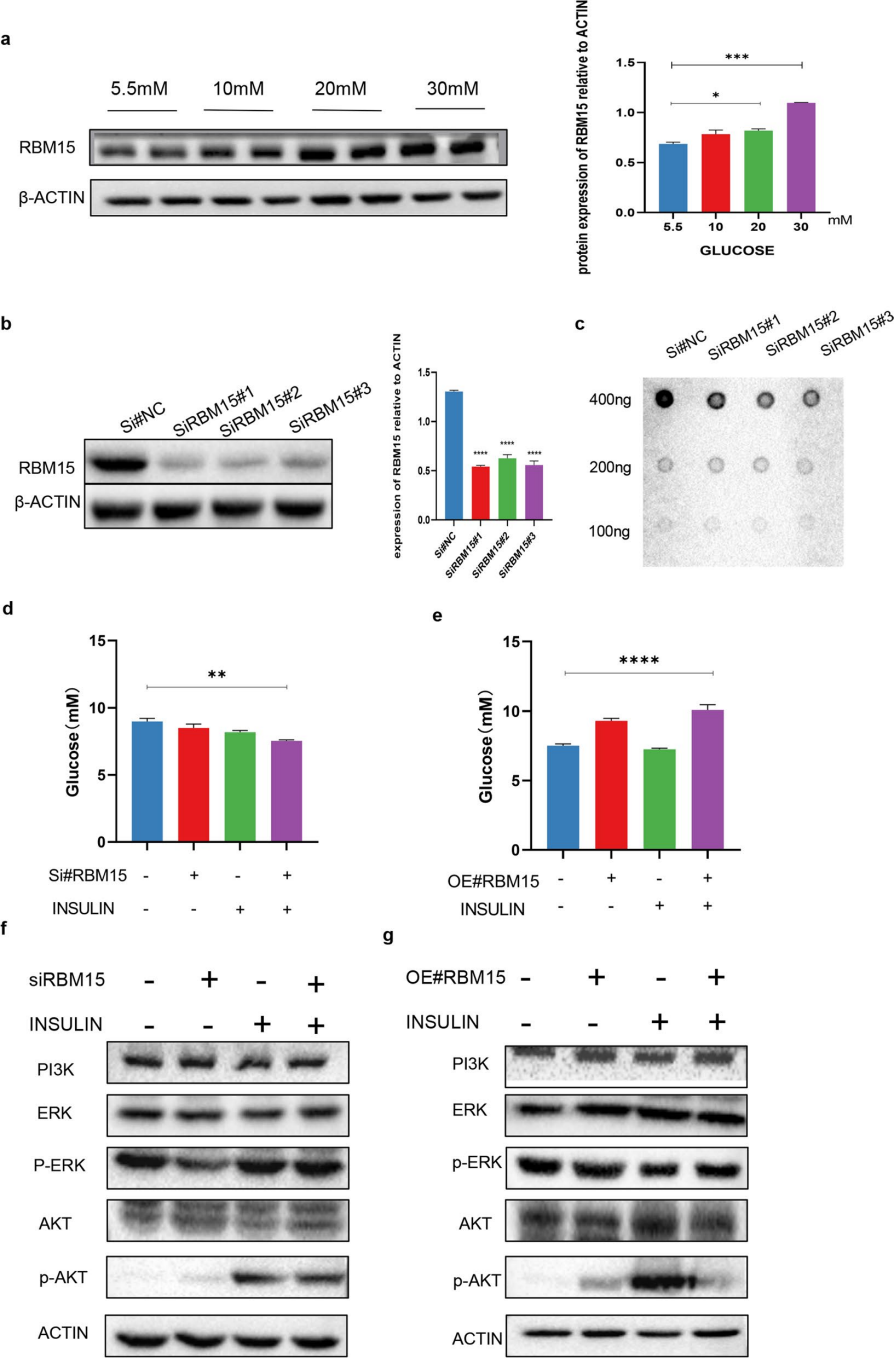
RBM15 enhances insulin resistance in vitro*.* **a** The expression level of RBM15 with the increase of glucose concentration. (*P < 0.05, ***P < 0.001 by ANOVA); **b** Western blot analysis to measure RBM15 protein levels in LO2 cells transfected with siNC and/or siRBM15. (****P < 0.0001 by ANOVA); **c** Global m6A level of RNA extracted from RBM15-knockdown LO2 cells was measured via m6A dot blot assays. RNAs were serially diluted and loaded equally with the amount of 400 ng, 200 ng and 100 ng. The intensity of dot blot represented the level of m6A modification. **d**, **e** Glucose uptake test of LO2 cells transfected with siRB15 or overexpression RBM15 and followed with or without 30-min insulin treatment. (**P < 0.01, ****P < 0.0001 by ANOVA); **f**, **g** LO2 cells were transfected with RBM15 siRNA or overexpression of RBM15 and followed with or without 100 nmol/L insulin stimulation for 30 min, PI3K, p-AKT, and p-ERK protein and their total protein levels were determined by Western blot

To investigate whether RBM15 affects the insulin sensitivity, an in-vitro experiment was performed. Firstly, the protein level of RBM15 increased proportionally with the glucose concentration gradient, reaching the highest level at 30 mM (Fig. 5a, Additional file 1: Fig. S3). Meanwhile, RBM15 was still highly expressed in situ treatment with high-glucose for 24 h and then with normal-glucose with 24 h and 48 h (Additional file 1: Fig. S4). Meanwhile, the global m6A methylation level also increased with the glucose concentration (Additional file 1: Fig. S6a). To further investigate the potential function of RBM15 in resistance insulin, we synthesized RBM15 specific siRNA to obstruct the expression of endogenous RBM15 in LO2 cell lines. RBM15 expression was reduced after siRBM15 transfection (Fig. 5b, Additional file 1: Fig. S6b). Dot blots assay was applied to examine the role of RBM15 in modulating m6A modification. Reduced expression of RBM15 led to decreased m6A levels (Fig. 5c, Additional file 1: Fig. S5). Moreover, glucose content in the supernatant of cells was reduced after knockdown of RBM15, whereas the opposite result was found after overexpression of RBM15(Fig. 5d, e). Furthermore, the silence of RBM15 in LO2 cells significantly enhanced the phosphorylation of key components of insulin signaling, indicating the increased insulin sensitivity by RBM15 silence (Fig. 5f, Additional file 1: Fig. S7a), while the overexpression of RBM15 exhibited the opposite results (Fig. 5g, Additional file 1: Fig. S7b). Moreover, the primary hepatocytes still revealed the similar results. The hepatocytes from GDM offspring demonstrated decreased insulin sensitivity than control (Additional file 1: Fig. S8). The above evidence suggests that RBM15 was highly expressed in hepatocytes under a hyperglycemic state, impeding the uptake of insulin and glycolysis.

### RBM15 regulates insulin sensitivity through m6A-guided inhabitation of CLDN4

**Fig. 6:**
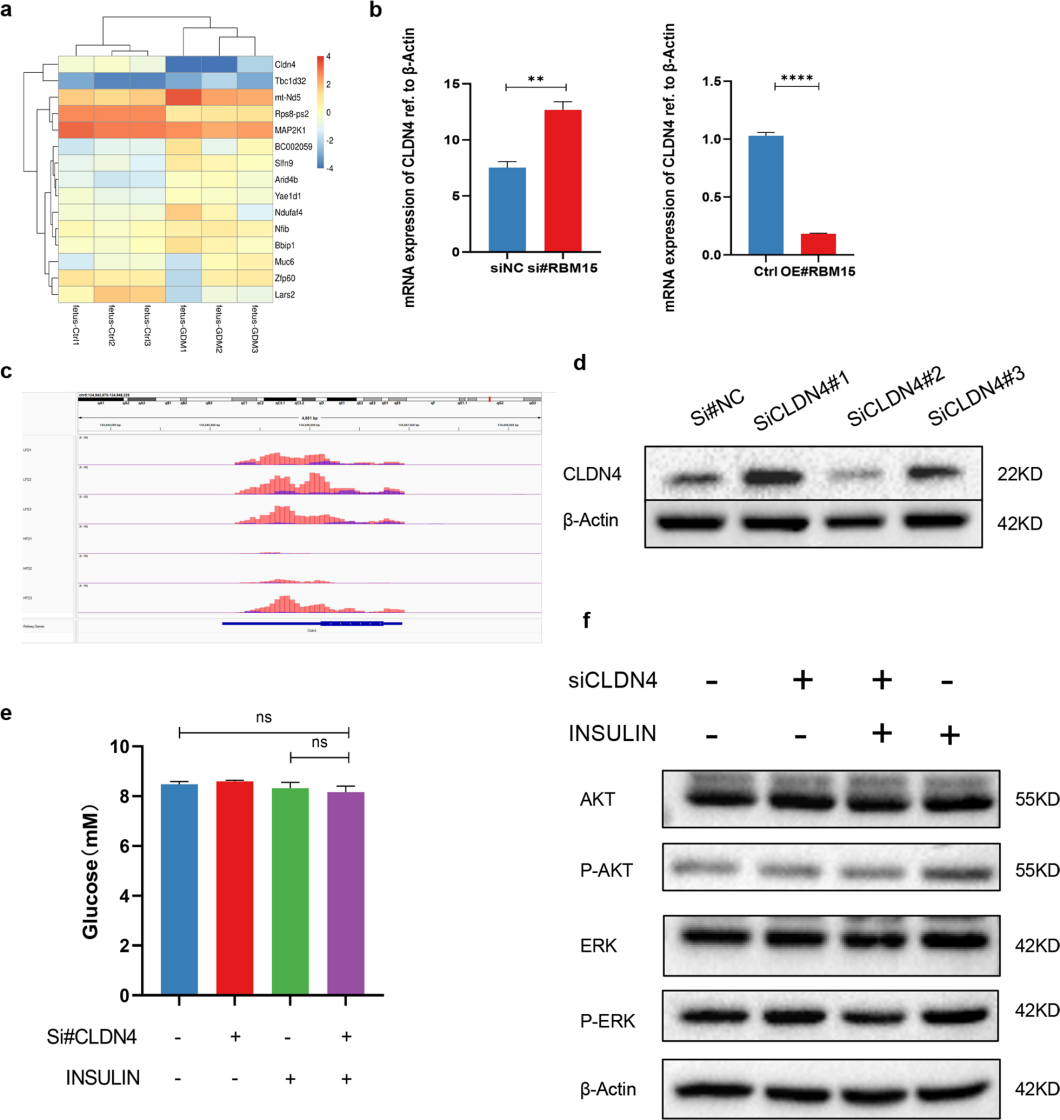
RBM15 regulates insulin sensitivity through m6A-guided inhabitation of CLDN4. **a** The heatmap of top 10 genes with differentially m6A peaks and differentially expression; **b** the mRNA of CLDN4 after knockdown and overexpression of RBM15; **c** IGV analysis for m6A abundance on CLDN4 mRNA in offspring liver of GDM or control; **d** the Merip-qPCR of CLDN4 after knockdown of RBM15; **e** glucose uptake test of LO2 cell lines transferred with siCLDN4 (P > 0.05 by ANOVA); **f** LO2 cells were transfected with CLDN4 siRNA followed with or without 100 nmol/L insulin stimulation for 30 min, PI3K, p-AKT, and p-ERK protein and their total protein levels were determined by Western blot

According to the MeRIP-seq and mRNA-seq, totally 201genes with differentially regulated m6A peaks and differentially expressed genes, we selected the top 10 genes (Fig. 6a) and tested the top 4 genes including *NIFD*, *Arid4b*, *Tbc1d32* and *CLDN4* after knockdown RBM15, CLDN4 was screened out that was the most differentially regulated genes (Fig. 6b, Additional file 1: Fig. SF9a–c). Meanwhile, the IGV analysis suggested that the m6A peaks of CLDN4 was remarkably increased in HFD group (Fig. 6c). We confirmed that the protein level of CLDN4 was down regulated in the liver of offspring (Additional file 1: Fig. SF10a), which was mainly located in cytoplasm and membrane according to the immunohistochemistry (Additional file 1: Fig. SF10b). To illustrate the role of CLDN4 in insulin sensitivity, we established CLDN4-knockdown cell lines (Fig. 6d). Although glucose uptake test showed no significant difference of glucose consumption after obstruction of CLDN4 (Fig. 6d), the classical phosphorylation of key components of insulin signaling was downregulated, indicating the decreased insulin sensitivity (Fig. 6e).

## Discussion

There are various chemical modifications of RNA, including m6A, m1A, m5C, and other methylation modifications, among which m6A modification is the most prevalent, abundant, and reversible methylation modification of both mammalian mRNA and non-coding RNA (Pinello et al. 2018, Cai et al. 2018, Roundtree et al. 2017). m6A modification plays an important role in many biological processes, such as regulation of mRNA stability (Huang et al. 2018), pre-mRNA splicing (Bartosovic et al. 2017), mRNA decay and translation (Shi et al. 2017), and DNA damage repair (Xiang et al. 2017). A series of studies have shown that m6A RNA modification is associated with various human diseases, including cancer, cardiovascular diseases (Paramasivam et al. 2020; Dorn et al. 2019), diabetes (De Jesus et al. 2019), preeclampsia (Gu et al. 2021) and recurrent abortion (Li et al. 2019). In this study, our data showed that m6A modification was increased in liver tissues of offspring born out of GDM, suggesting that m6A modification might develop insulin resistance and metabolic syndrome of the fetus.

Over the past decade, Lepr/+ mice have been used as a model of GDM because of accordance with the phenotype of GDM during pregnancy, such as reduced glucose tolerance, insulin tolerance, and elevated weight gain. However, some researches using this model have found that this phenotype cannot be simulated (Talton et al. 2016; Plows et al. 2017). The literature review found that mice fed with HFD before pregnancy showed phenotypes such as hyperglycemia and insulin resistance at late-stage of pregnancy. Thus, in our study, mice fed with HFD were used to establish the GDM model. Significant differences of glucose intolerance and insulin sensitivity were exhibited at the middle and late stage of pregnancy, suggesting the successful establishment of HFD-induced GDM model.

Interestingly, the weight of fetus from GDM group was slightly higher than the control group, nonetheless, the weight of the offspring in both groups exhibited indifferent as the week-age increased, suggesting that changes in the intrauterine microenvironment may contribute to neonatal macrosomia, but did not appear to be decisive for fetal growth and development. The GDM offspring mice exhibited poor glucose intolerance and insulin sensitivity, combined with plasma insulin concentration and HOMA-index, intrauterine hyperglycemia probably has a long-term impact on the offspring's blood glucose level and metabolism, resulting in susceptibility to develop insulin resistance.

RBM15 belongs to the SPEN (Split-end) protein family involved in cell fate decision (Hiriart et al. 2005). It is mainly located in the nucleus, which might interact with spliceosome components. RBM15 was initially identified as a leukemic ectopic which participates in pediatric acute megakaryocytic leukemia (Xiao et al. 2012; Ayllon et al. 2017). Notably, RBM15 may play different roles in different diseases. A previous study has shown that RBM15 regulates the alternative splicing of Mpl-TR, which may impair the hematopoietic stem cells engraftment (Xiao et al. 2015). Another study has shown that RBM15 mediated the degradation of BAF mRNA via the mechanism of mRNA methylation in the developing cortex (Xie et al. 2019). Moreover, RBM15 has been reported to promote the occurrence and development of laryngeal squamous cell carcinoma by maintaining the stability of TMBIM6 (Wang et al. 2021). In this study, our data firstly revealed that RBM15 might contribute to the development of metabolic syndrome based on insulin resistance through the m6A-mediated regulation of CLDN4 in GDM offspring.

It is worth noting that the downstream of RBM15 was vastly studied over the years, but its upstream roles remain poorly understood. A recent study showed that AS-RBM1, AS and lncRNA, can regulate the differentiation of megakaryocytes by regulating the expression level of RBM15 (Tran et al. 2016). Similarly, microribonucleic acid (miRNA) can inhibit the activity of several m6A methylation enzymes (Cai et al. 2018); thus, whether miRNA affects the expression of RBM15 in this study is still worthy of further exploration.

Most studies have shown that m6A modification plays a role in metabolism, obesity (Sun and Zhang 2021), type II diabetes (Yang et al. 2019), and cardiovascular disease (Zhong et al. 2020) by regulating glucose/lipid metabolism and inflammatory/immune response. Furthermore, according to Wang et al. (2020), m6A modification plays an important role in driving functional maturation of neonatal islet beta cells. According to our results, although there was no abnormality of the fetus at infant stage, the metabolic organ of GDM offspring mice at adult stage lost its normal structure with clearly visible vacuoles and exhibited obvious glycose deposition, indicating that intrauterine hyperglycemia might have an effect on the metabolic level of the progeny at adult stages. Despite the phenotype of metabolic change, parts of metabolites still showed difference including saturated fatty acids, which implied that the ability of glucolipid metabolism in offspring mice might be defective to some extent. Combined with above results, intrauterine hyperglycemia might affect fetal epigenomic modification; however, due to immature fetal body development, the regulated genes may not temporarily play a role, or short-term changes will not have a significant impact on fetal metabolism. As the offspring grows, epigenetic genes may become more functional, leading to metabolic disorders in adolescence and adulthood. Further exploring the specific mechanism of how m6A regulates the metabolic changes in GDM offspring mice is the future direction of our research team.

## Conclusion

Overall, our study has demonstrated that the offspring of GDM mom is much easier to develop metabolic disorders in which RBM15 might play an important role. Overexpression of RBM15 suppresses hepatic insulin sensitivity via m6A-guided regulation. Moreover, we characterized the differential m6A methylom between the GDM fetal liver and control, suggesting a potential association between m6A methylation and the regulation of glycolipid metabolism in the liver, therefore providing underlying basis of metabolic disorders of GDM offspring for future researches.

## Methods

### Mice

Twenty-four 8–12-week-old female C56BL/6J mice weighing 20–25 g from the Experimental Animal Center of Chongqing Medical University were randomly divided into two groups: GDM group and control group. GDM group was fed with high-fat diet (Research Diets D12451) for 1 week to mating and throughout pregnancy, while control group was fed with normal diet (Research Diet 1022). Then the two groups were mated with age-matched male mice. The presence of a vaginal plug was considered gestational day (GD) 0.5. Oral glucose tolerance test (OGTT) and insulin tolerance test (ITT) were performed at GD0.5, GD11.5, and GD16.5. Six mice of each group were killed at GD18.5 to collect the liver tissue of the fetus. The remaining mice were left to delivery. All female offspring were selected for this study. Fetus weaning was done and then was separately fed with a standard diet for 12 weeks. OGTT and ITT were performed at 12 weeks. Then F1 generation was killed to collect serum and liver tissue.

### Oral glucose tolerance test and insulin tolerance test

After inducing fasting for 12 h, a fasting blood glucose level was determined via the caudal vein of the mice and recorded as blood glucose for 0 min. For OGTT, the mice were administered with 20% glucose solution (2 g/kg body weight) via intragastric gavage. For ITT, the mice were administered with insulin (2.5 U/kg body weight) via intraperitoneal injection. Random blood glucose levels were determined at 30 min, 60 min, 90 min, and 120 min by a glucometer (Nova StatStrip Xpress; Nova Biomedical, Waltham, UK). According to the measured concentrations, the area under the curve (AUC) was calculated to compare the difference between the groups.

### Enzyme-linked immunosorbent assay (ELISA) and HOMA-index

Insulin concentration in serum was measured with Mouse Insulin (INS) ELISA Kit (Cusabio), Cholesterol and Triglyceride was measured by Mouse Cholesterol ELISA Kit (JINGMEI) and Mouse Triglyceride ELISA Kit (JINGMEI) respectively. Liver tissue lysates or serum were then subjected to the assay according to the manufacturer’s instructions.

$$\text{HOMA-index}=\text{fasting glucose concentration} * \text{fasting insulin concentration}/22.5$$

### Quantitative RT-PCR

Total RNA of cultured cells and liver tissue was extracted using Trizol (Invitrogen, Carlsbad, CA, USA), following manufacturer instructions. The RNA concentration was measured with a NanoDrop 2000 (Thermo Fisher Scientific). Complementary DNA (cDNA) was prepared with PrimeScript RT reagent Kit with gDNA Eraser (MCE). qRT-PCR was performed using Green SYBR Premix Ex Taq II (MCE) in CFX96 Real-Time System (Bio-Rad). GADPH normalized relative gene expression for methyltransferase differentiation using a 2−ΔΔCt method. The primers are listed in Table 1.

**Table 1:** Primer sequence

Gene name		Forward	Reverse
*RBM15*	House mouse	TTCACGAGTTCAAACGCTTCG	ACAAAGGCTACCCGCTCATC
*METTL3*	House mouse	GAGTTGATTGAGGTAAAGCGAGG	GGAGTGGTCAGCGTAAGTTACA
*IGF2BP1*	House mouse	AGCAGACCAGGCAAGCTATC	CCCATTCTCAGGACCTTGCG
*IGF2BP2*	House mouse	GTCCTACTCAAGTCCGGCTAC	CATATTCAGCCAACAGCCCAT
*GAPDH*	House mouse	AGGTCGGTGTGAACGGATTTG	TGTAGACCATGTAGTTGAGGTCA
*CLDN4*	Human	TCTCCTCTGTTCCGGGTAGG	CGTCCATCCACTCTGCACTT

### Western blot

Proteins were extracted from liver tissues and LO2 cells using RIPA lysis buffer supplemented with PMSF (Beyotime Institute of Biotechnology, China). Protein samples were electrophoresed, then transferred onto PVDF membranes (MilliporeSigma, USA), and blocked with 5% nonfat milk at room temperature for 1 h. Then the PVDF membrane was incubated with the primary antibody solution anti- RBM15 antibody (1: 1000, Proteintech, China),anti-PI3K antibody (1: 1000; CST, USA),anti-ERK antibody (1:1000, CST, USA), anti-p-ERK antibody (1:1000, Wanlei, China), anti-AKT antibody (1:1000; Proteintech, China),anti-p-AKT antibody (1:1000; Proteintech; China) and anti-β-ACTIN (1:1000; Servicebio, China) overnight at 4 °C. Monoclonal anti-rabbit IgG (1:5000, ZSGB-Bio, China) or monoclonal anti-mouse IgG (1:5000, ZSGB-Bio, China) were incubated with the PVDF membrane for 1 h at room temperature. Finally, enhanced chemiluminescent substrate (MilliporeSigma, USA) was used to detect blots by a ChemiDoc XRS + system (Bio-Rad, USA).

### Measurement of the global m6A level

Total RNA of the liver was extracted as above and subjected to m6A measurement using an m6A RNA Methylation Assay Kit (Abcam, USA) according to the manufacturer’s manual. In brief, RNA solution (200 ng, 8 µL), negative control, and standard samples were coated onto the strip wells with binding solution and incubated at 37 °C for 90 min. Then, the capture antibody and detection antibody solutions were added to the wells and incubated at room temperature for 30 min and 60 min respectively in order. The global m6A level was measured by reading the absorbance at 450 nm using microplate reader.

### MeRIP-sequencing

MeRIP-sequencing and following data analyses were mainly supported by LC-BIO Bio-tech. (Hangzhou, China). Total RNA of the liver of two groups was isolated as before. 50 µg of total RNA was required to obtain poly(A) mRNA (Invitrogen, USA). The fragmentation reagent was added to fragment mRNA to about 100 nt. The fragmented RNA was sorted into two portions. To one, immunomagnetic beads with premixed m6A antibody (Synaptic Systems, Germany) were added to enrich the mRNA fragments containing m6A methylation. The other was used as a control. Paired-end 2 × 150 bp sequencing was performed on the Illumina Novaseq 6000 platform of LC-BIO Bio-tech. (Hangzhou, China). Raw data was cleaned using Fastp software (https://github.com/OpenGene/fastp). HOMER was used to predict the m6A hot motifs. Then, expression levels for all mRNAs from input libraries were calculated by StringTie.

### Cell culture

The human liver cell line LO2 was obtained from the Cell Bank of Type Culture Collection of Chinese Academy of Sciences, Shanghai Institute of Cell Biology. These cells were cultured with DMEM/F12(Gibco, China) routinely supplemented with 10% fetal bovine serum (FBS, PAN3000), penicillin (100 units/mL), and streptomycin (100 μg/mL) and incubated at 37 °C in 5% CO2 humidified air. siRNA was transfected into LO2 cells with Lipofectamine 2000 (Thermo Fisher, CA, USA). SiRBM15#1: 5′-GCCUGUUUCAUGAGUUCAATT-3′; siRBM15#2: 5′-GCAUACAGUCUUGAGCCAATT-3′; siRBM15#3: 5′-GCCUCUGCCCUUGACUCAUTT-3′.

### Primary hepatocytes isolation and culture

Primary hepatocytes were isolated from the 6-week-old offspring mice by sequential in perfusion with 50 mL of wash buffer I and then with 30 mL of 0.05% collagenase IV (Solarbio, China) solution. Cells were collected after centrifugation at 50*g* for 3 min at 4 ℃. Cell viability was determined by the trypan blue exclusion method. Primary hepatocytes were plated into six-well plates at a density of 2.5 × 106 cells/well. Primary HCs were cultured in Williams' Medium E (Gbico, USA) containing 10% FBS and 1% penicillin/streptomycin (Gibco) and incubated at 37 °C in 5% CO2 humidified air.

### m6A dot blot assay

Total RNA isolated from LO2 cells was denatured at 95 °C for 5 min. Samples (400 ng, 200 ng, and 100 ng) were deposited on a Hybond-N+ membrane (Millipore, USA), the membrane was cross-linked by UV light for 5 min and the membrane was incubated with m6A antibody (1:5000, Synaptic System, Germany) overnight at 4 °C. After incubating with secondary antibody for 1 h at room temperature, dot blots were imaged by the ChemiDoc XRS + system (Bio-Rad, USA).

### Immunohistochemistry

Liver tissue blocks were fixed with 4% paraformaldehyde overnight and embedded in paraffin. A standard immunohistochemistry staining procedure was used: deparaffinization in xylene, dehydration in a serial ethanol gradient, quenching of endogenous peroxidase activity with H2O2 followed by blocking nonspecific binding with goat serum, and then microwaved in 10 mM citric sodium for 15 min to retrieve antigens. Then the sections were incubated with anti-RBM15 primary antibody (1:100, Proteintech, China) at 4 °C overnight, followed by incubation with secondary antibody conjugated with horseradish peroxidase for 60 min at room temperature.

### Gas chromatography-mass spectrometry (GC–MS)

The metabolites were extracted using a methyl chloroformate (MCF) derivatization means based on the protocol published by Smart et al. (2010). The derivatized metabolites were analyzed in an Agilent 7890B Gas Chromatograph coupled with a MSD5975 mass selective detector (Agilent, USA) emitting electrons at 70 eV. Automated mass spectral deconvolution and identification system (AMDIS) was used to identify the GC–MS chromatographic peaks. The peaks were identified based on two standers; > 85% match to the fatty acid library spectra and within a 1-min bin of the library chromatographic retention time. The relative abundance of metabolites were quantified via our in-house MassOmics that uses the highest reference of ion fragments.

### Statistical analyses

Statistical data were analyzed by Student’s t test (2 groups) and ANOVA test (more than 2 groups) using Prism 8 software (GraphPad Software, USA). P value ≤ 0.05 was considered as a significant difference, *P < 0.05, **P < 0.01, ***P < 0.001, ****P. < 0.0001.

## Supplementary Information

**Additional file 1: Figure S1.** (a) The cholesterol concentration in liver tissue of 12-week-old offspring; (b) The cholesterol concentration in serum of 12-week-old offspring; (c) The triglyceride concentration in liver tissue of 12-week-old offspring; (d) The triglyceride concentration in serum of 12-week-old offspring. **Figure S2.** (a) Original Western blots of Fig. 3g. (b) Original Western blots of Fig. 3h. **Figure S3.** Original Western blots of Fig. 5a. **Figure S4.** The RBM15 protein level of LO2 cells after treated with high-glucose for 24 h and then with normal-glucose for 24 h and 48 h. **Figure S5.** Original Western blots of Fig. 5b. **Figure S6.** (a). the global m6A level with the gradient of glucose concentration; (b) Original dot blots of Fig. 5c. **Figure S7.** (a) Original Western blots of Fig. 5f. (b) Original Western blots of Fig. 5g. **Figure S8.** Insulin sensitivity of primary hepatocytes from the GDM offspring and control group. **Figure S9.** (a) mRNA expression of NIFD after knockdown of RRBM15; (b) mRNA expression of Arid4b after knockdown of RRBM15; (c) mRNA expression of Tbcld32after knockdown of RRBM15. **Figure S10.** (a) The protein level of CLDN4 in offspring liver of GDM and control; (b) immunohistochemistry of CLDN4 for the liver of GD18.5 fetus and 12-weeks offspring.

## Data Availability

The data and materials of this study are available from the corresponding authors for reasonable requests.

## Abbreviations

GDM: Gestational diabetes mellitus
m6A: N6-methyladenosine
OGTT: Oral glucose tolerance test
ITT: Insulin tolerance test
GD: Gestational days
MeRIP-seq: Methylated RNA Immunoprecipitation sequencing
mRNA-seq: MRNA sequencing
IHC: Immunohistochemical
RBM15: RNA binding motif protein 15
METTL3: Methyltransferase-like 3
IGF2BP1: Insulin like growth factor 2 mRNA binding protein 1
IGF2BP2: Insulin like growth factor 2 mRNA binding protein 2
CLDN4: Claudin 4
WTAP: Wilms Tumor 1-associated protein
METTL14: Methyltransferase-like 14
HFD: High-fat diet
LFD: Low-fat diet
AUC: Area under curve
CBLL1: Cb1 proto-oncogenes like 1
ZC3H13: Zinc-finger CCCH-type containing 13
VIRMA: Virilizer like m6A methyltransferase associated protein
FTO: Fat mass and obesity-associated protein
ALKBH5: ALKB homologous 5
YTHDF: YTH N6-methyladenosine RNA binding protein
eIF3: Eukaryotic translation initiation factor 3
HOMA-IR: Homeostasis model assessment-Insulin Resistance
NIFD: Nitrogenase molybdenum-iron protein alpha chain
Arid4b: AT-rich interaction domain 4B
Tbcld32: TBC1 domain family member 32
