# Supplementary material for: RBM15 suppresses hepatic insulin sensitivity of offspring of gestational diabetes mellitus mice via m6A-mediated regulation of CLDN4
Source: Mol Med. 2023 Feb 20;29:23. doi: 10.1186/s10020-023-00615-8 (PMC9942341; doi:10.1186/s10020-023-00615-8)
Supplement: Supplementary file 1 — Additional file 1: Figure S1. (a) The cholesterol concentration in liver tissue of 12-week-old offspring; (b) The cholesterol concentration in serum of 12-week-old offspring; (c) The triglyceride concentration in liver tissue of 12-week-old offspring; (d) The triglyceride concentration in serum of 12-week-old offspring. Figure S2. (a) Original Western blots of Fig. 3g. (b) Original Western blots of Fig. 3h. Figure S3. Original Western blots of Fig. 5a. Figure S4. The RBM15 protein level of LO2 cells after treated with high-glucose for 24 h and then with normal-glucose for 24 h and 48 h. Figure S5. Original Western blots of Fig. 5b. Figure S6. (a). the global m6A level with the gradient of glucose concentration; (b) Original dot blots of Fig. 5c. Figure S7. (a) Original Western blots of Fig. 5f. (b) Original Western blots of Fig. 5g. Figure S8. Insulin sensitivity of primary hepatocytes from the GDM offspring and control group. Figure S9. (a) mRNA expression of NIFD after knockdown of RRBM15; (b) mRNA expression of Arid4b after knockdown of RRBM15; (c) mRNA expression of Tbcld32after knockdown of RRBM15. Figure S10. (a) The protein level of CLDN4 in offspring liver of GDM and control; (b) immunohistochemistry of CLDN4 for the liver of GD18.5 fetus and 12-weeks offspring. [file 10020_2023_615_MOESM1_ESM.docx]

**Additional file**


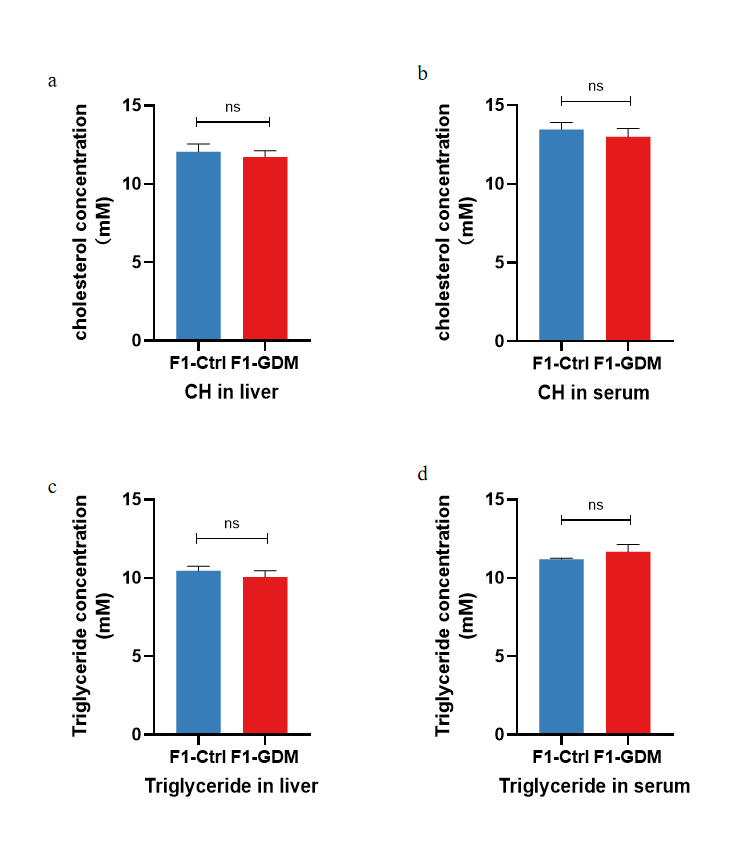


**Figure S1:(a) The** **cholesterol concentration in liver tissue of 12-week-old offspring;(b) The cholesterol concentration in serum of 12-week-old offspring;(c) The** **triglyceride concentration in liver tissue of 12-week-old offspring;(d) The triglyceride concentration in serum of 12-week-old offspring.**

**
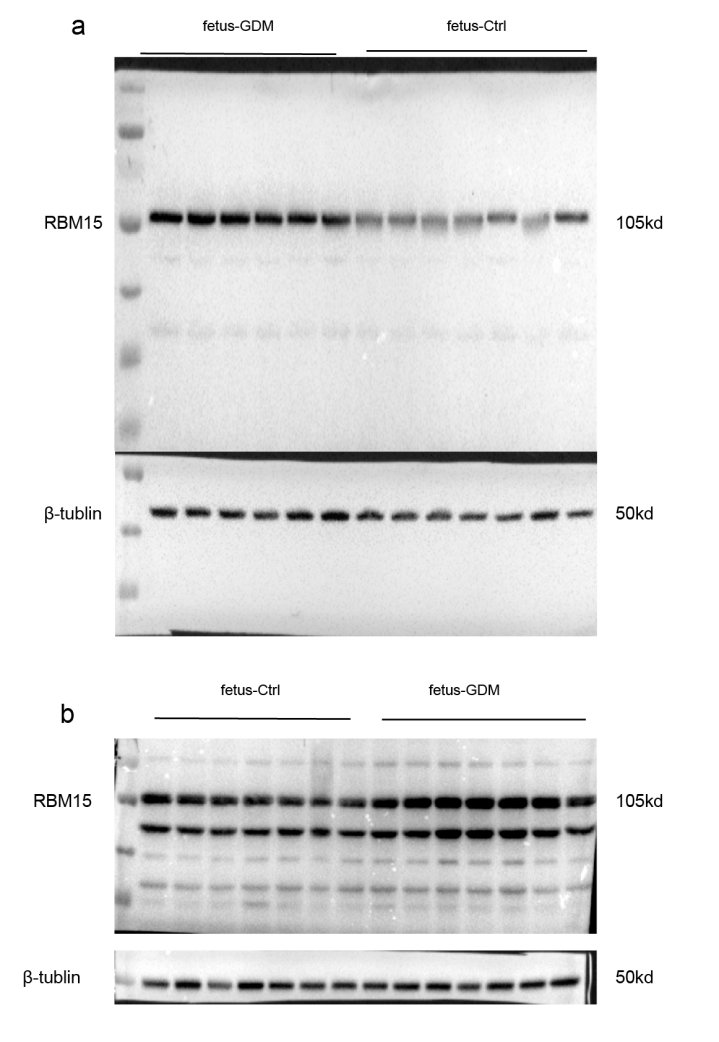
**

**Figure S2: (a) Original Western blots of Figure 3g. (b) Original Western blots of Figure 3h.**

**
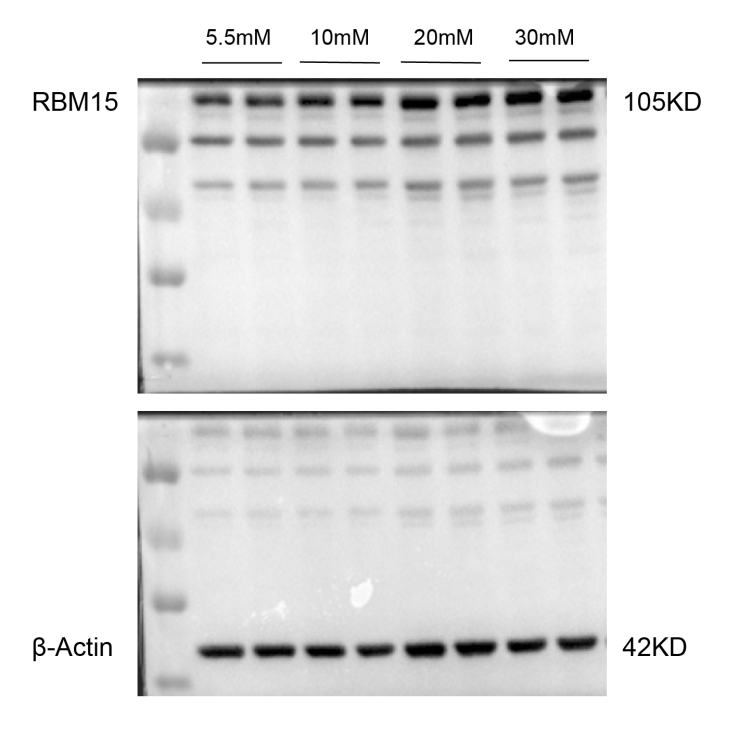
**

**Figure S3: Original Western blots of Figure 5a.**


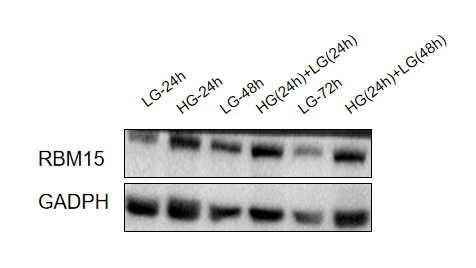


**Figure S4: The RBM15 protein level of LO2 cells after treated with high-glucose for 24h and then with normal-glucose for 24h and 48h.**

**
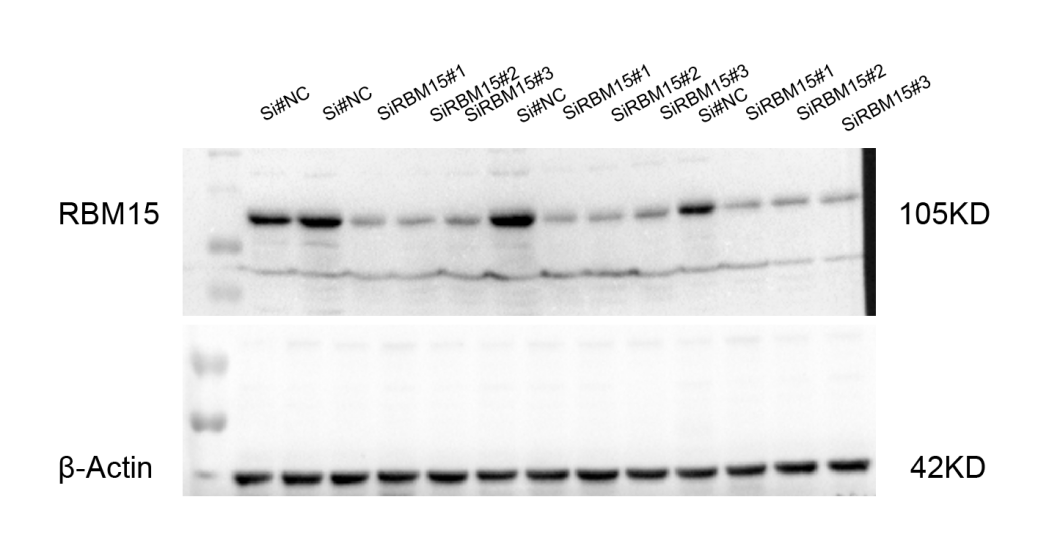
**

**Figure S5: Original Western blots of Figure 5b.**


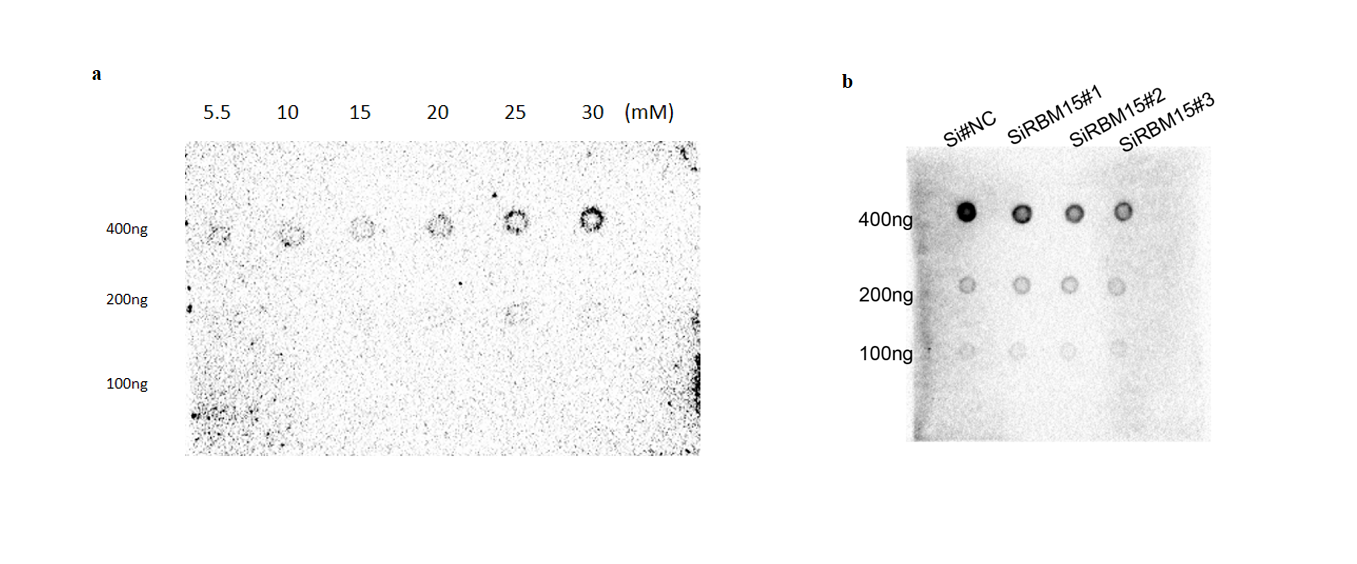


**Figure S6：(a). the global m6A level with the gradient of glucose concentration; (b) Original dot blots of Figure 5c**

**
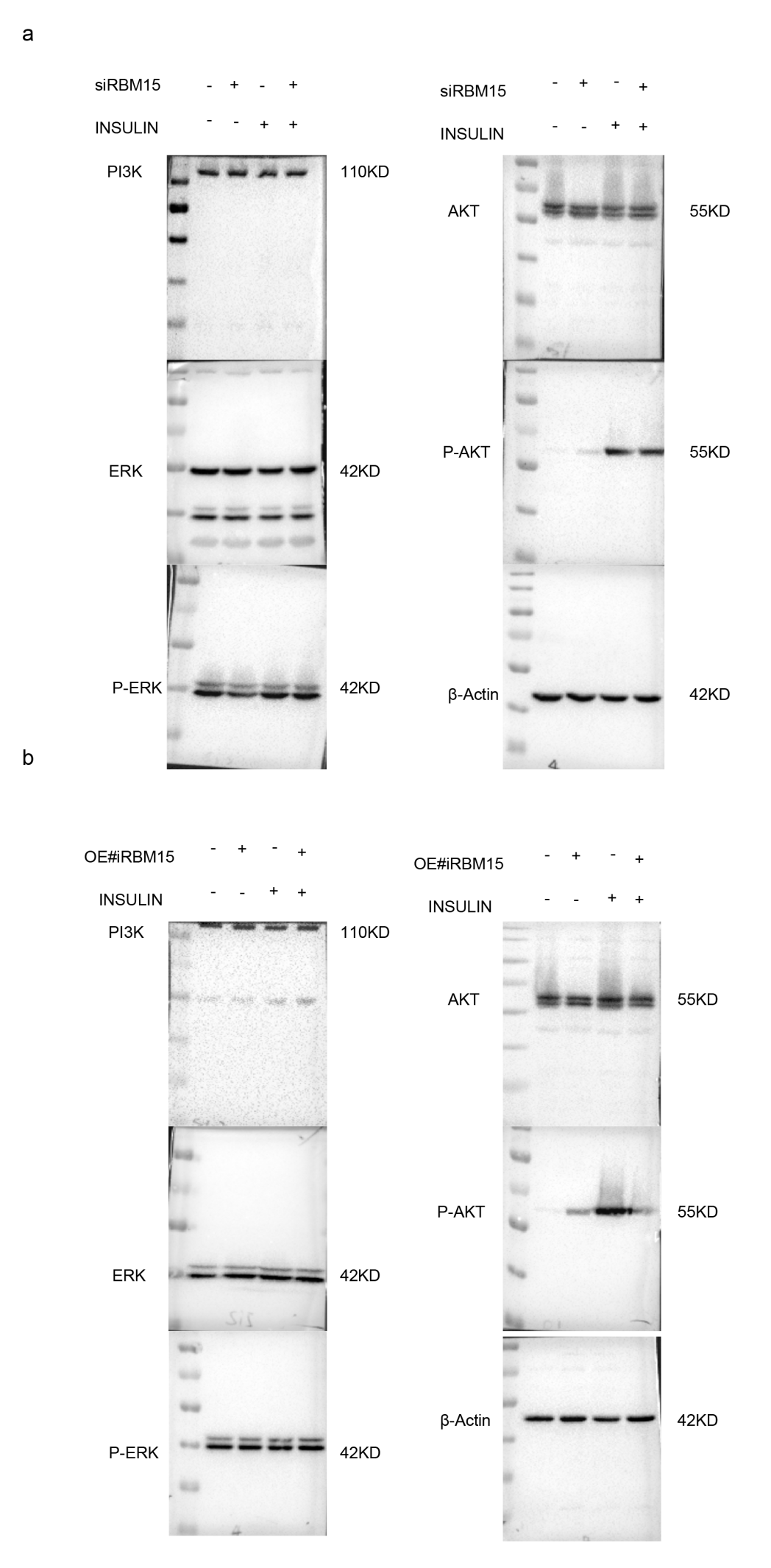
**

**Figure S7: (a)Original Western blots of Figure 5f. (b) Original Western blots of Figure 5g.**


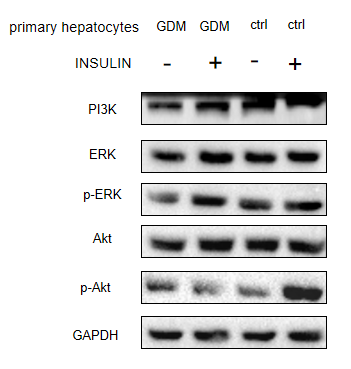


**Figure S8: insulin sensitivity of primary hepatocytes from the GDM offspring and control group.**


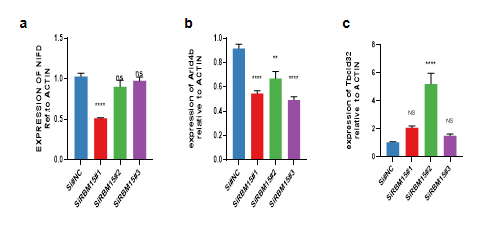
 **Figure S9: (a)mRNA expression of NIFD after knockdown of RRBM15;(b) mRNA expression of Arid4b after knockdown of RRBM15; (c)mRNA expression of Tbcld32after knockdown of RRBM15.**


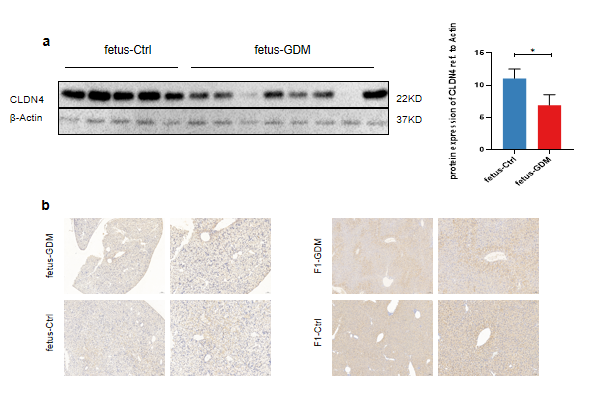


**Figure S10: (a) The protein level of CLDN4 in offspring liver of GDM (n=8) and control (n=5); (b) immunohistochemistry of CLDN4 for the liver of GD18.5 fetus and 12-weeks offspring.**
